# Factors Associated With Testicular Cancer Risk Among New American Military Recruits During Wartime

**DOI:** 10.1177/15579883261433806

**Published:** 2026-03-15

**Authors:** DA Nelson, OT Hill, SA Skaggs, WJ Brown

**Affiliations:** 1 School of Health Professions, University of Texas Health Science Center at San Antonio, San Antonio, TX, USA; 2 Martin Army Community Hospital, Fort Benning, GA, USA

**Keywords:** testicular cancer, oncology/cancer, occupational health, epidemiology of men’s health, general health and wellness, sexual health, sexuality

## Abstract

Testicular cancer (TCa) comprises the majority of peri/postpubertal male neoplasms and has increased in incidence. A review of the literature finds little information on short-term risks associated with mens’ occupational choices. The U.S. Army includes a large population in the age range of highest TCa risk. We examined whether combat duty and occupational categories were associated with TCa in this population during a recent period of prevalent combat operations. We employed official medical, administrative and sociodemographic data on 210,483 males new to active-duty service in the U.S. Army during 2011–2014, when combat operations in Afghanistan continued apace and those in Iraq were waning. We calculated TCa diagnosis incidence rates by occupation and service time, compared predictor distributions, and devised a multivariable survival regression model. There were 44 new recruits diagnosed with TCa (period prevalence: 0.02%; incidence rate: 11.61 per 100,000 person-years). Within up to 4 years of service, the adjusted TCa diagnosis risk increased twofold for those in the infantry occupation when compared with other military occupations (*p* = .045) but was not associated with combat deployment. Adjusted TCa risks increased with age and White race and decreased with service time. Variation in adjusted TCa diagnosis risk by occupation was seen within 4 years following service entry. This variation could have potentially occurred due to selection effects for which we could not control, yet causative occupational exposures cannot be ruled out with our data. The results suggest the need for further research to identify causal pathways and risk mitigation strategies.

## Introduction

Detrimental effects on health possibly associated with military service and combat duty in particular (Bethmann & Cho, 2023; National Library of Medicine, 2024) could factor into whether young people today consider the military to represent a plausible career option (Berkeley Political Review, 2024). When considering the top, serious physical health problems of young men, testicular cancer (herein, “TCa”) deserves attention with respect to occupational risks. TCa comprises the majority of peri- and postpubertal male neoplasms (Batool et al., 2019) and males from puberty to age 44 are at the highest age-based risk of TCa (Palumbo et al., 2019).

Members of this demographic group represent primary targets of military recruiting. Young adult males thus comprise the vast bulk of the U.S. military (U.S. Defense Manpower Data Center, 2025). Consistent with the military male population’s demographic makeup, TCa is the most common cancer type among U.S. military men (Armed Forces Health Surveillance Center, 2016).

While overall mortality rates due to TCa declined after 1970 due to improving treatments, upward TCa rate trends were noted thereafter (Bosl & Motzer, 1997). TCa cases increased from 5.4 to 6.0 cases per 100,000 men per year from 2000 to 2018 alone (National Cancer Institute, 2024). The increasing TCa rate means that more men are experiencing the stress, uncertainty, and potential for fertility loss associated with a disorder for which orchiectomy is a standard treatment (Parekh et al., 2020; Stephenson et al., 2024).

In light of these statistics, identifying occupational TCa predictors appears more important than ever. Is it fundamentally realistic that occupational choices including military service could influence TCa risk? Most TCa risk may be established in utero (Giona, 2022), possibly limiting the contribution of occupational choices.

Inherited TCa risk factors include cryptorchidism, family history, and above-average adult height along with prior contralateral TCa (Batool et al., 2019; Gilligan et al., 2019; Giona, 2022; Herr et al., 2022). If environmental factors including occupational exposures were substantial TCa predictors, then variation in TCa risk might be seen across identifiable populations, such as by location. A large case–control study of TCa in Europe (*N* = 3,297 cases) did not reveal systematic risk differences associated with geographic locations (Sloan et al., 2015), suggesting the importance of factors beyond location.

The increased rate of TCa seen in young, working-age men has led to speculation that occupational exposures could be more important than is realized (Brenner et al., 2019; Faja et al., 2022; Mester et al., 2010; Shankar et al., 2020; Yousif et al., 2010). “Forever chemicals,” certain pharmaceuticals, and pesticides have generally been associated with TCa (Faja et al., 2022), and forever chemicals have been specifically associated with TCa among military men (Purdue et al., 2023). Studies of military men have also found increased TCa risks associated with nonionizing radiation exposure (Yousif et al., 2010) and service in certain aviation occupations (Webber et al., 2022). The need for more research into TCa predictors in military populations has been noted (Webber et al., 2022).

Latency periods present one challenge to studying whether military or other occupational exposures constitute TCa risk factors. Latency periods across broad cancer categories range widely (Berrington de Gonzalez et al., 2012; Howard, 2015). Peer-reviewed evidence on latency with respect to occupational exposures and TCa appears scant, raising a fundamental question: Is it plausible that TCa risk could vary in a detectable fashion across occupations relatively soon after entering them? If so, new evidence could lead to risk mitigation and screening improvements.

As the largest U.S. military branch (U.S. Department of Defense [DoD], n.d.), the U.S. Army represents a logical target for the study of these ideas. In addition to the population size, the Army is subject to standardized medical and administrative information capture in digital records that have proven useful in large studies involving causal inference (Nelson & Kurina, 2013; Nelson et al., 2017). The Army’s soldiers receive universal free health care (Tricare, 2022) and undergo annual, pre- and post-combat, and training-related health screenings (U.S. Army, 2019, n.d.-a; U.S. Military Health System, 2023).

Overseas military combat duty carries the potential for exposure to a wide range of adverse exposures including chemicals, metals, radiation, airborne toxins from “burn pits,” and contaminated water (Gidley, 2022; National Library of Medicine, 2024). Combat duty arguably represents the most intensive military experience after which men could face adverse health effects. Our intent was to explore associations between incident TCa and two main factors of interest following initial entry to service: combat duty and military occupations. We therefore employed data on the U.S. Army from a recent era during which combat duty was prevalent. The methods were configured to account for time in military service and other possibly predictive or confounding factors.

## Method

### Dataset and Study Population

We leveraged data extracts from official personnel and health records of men who served on active duty with the U.S. Army. A central goal was to capture subjects with contemporary or recent combat exposures versus the observation period. We therefore used data from 2011 to 2014, a time in which the full range of military exposures including combat duty was applicable. Intensive combat operations in southwest Asia proceeded apace at that time, primarily in Afghanistan (U.S. Congressional Research Service, 2019). Members of the Army who served during this era underwent a mean of 1.6 combat deployments per person in total (Committee on the Assessment of the Readjustment Needs of Military Personnel, Veterans, and their Families, 2013).

To enable detection of incident TCa, we confined the study population to new recruits who entered service during the data period. This study population could be expected to lack known cancer histories and possess intact sex organs due to U.S. military recruitment standards and standard medical practice. Service entry is prohibited if the applicant has a history of malignant tumors or an absence of one or both testicles (U.S. Army, 2019), and orchiectomy of the affected testes with dissection of adjacent tissues is standard treatment in most TCa cases (Stephenson et al., 2024). The result is that new Army recruits arguably share a comparable baseline status for subsequent incident TCa case detection.

The normal maximum age at enlistment in the U.S. Army during the time of our data was 42 years (Haberman & Pollard, 2023). Exceptions could be made per the Army’s waiver system in circumstances, such as if medical histories were adverse, but applicant qualifications were otherwise needed (Pollard et al., 2022). We limited the study to the regularly recruited population who entered military service at age 42 or younger. We constructed a longitudinal “panel” data structure representing their active military service time per the personnel records. All data used in the analyses were de-identified to the “limited” dataset definition (Office for Civil Rights, 2024).

### Dependent Variable

We used two sources to identify incident TCa diagnoses and devise the study’s dependent variable. First, we leveraged official health data from the U.S. Military Health System Data Repository that captures details of care rendered under military health benefits (U.S. Military Health System, 2019). These data provided diagnosis codes recorded in out- and inpatient care per the International Classification of Disease, Ninth Revision, Clinical Modification (ICD-9-CM) system in use at the time. The endpoint was defined upon the assignment of a diagnosis code beginning with “186” (malignant neoplasm of testis) as used in other published research on TCa (van der Meer, 2024).

It was theoretically possible that TCa diagnoses could be documented outside of these records and escape our visibility. For example, a soldier could choose to receive care under a civilian spouse’s separate health coverage. Wherever care is received, active-duty soldiers with major diagnoses involving work excusals for care or recovery are required to have these excusals formally documented. Such documentation occurs using standardized forms on which clinicians must describe the associated medical diagnoses (U.S. Army, 2019).

After reflection on these ideas, we additionally leveraged the Army’s database of work excusals (Smith, 2015) as our second diagnosis source to provide additional surveillance of TCa diagnoses. We assigned a dichotomous value at the appropriate time in affected subjects’ panels to identify TCa documented under either source. We followed subjects for such diagnoses until the end of person-specific data due to service discharge, the end of the total data in December 2014, or a TCa diagnosis, whichever occurred first.

### Independent Variables

We employed data derived from official personnel records to organize a manageable complement of predictor variables. Our goal was to address the main factors of interest including occupations and combat experience while controlling for plausible confounders. We sought to maximally include potentially useful predictors without “overfitting” regression analyses by including excessive independent variables versus the available outcomes. Overfitting is a risk in the case of a relatively uncommon outcome like TCa.

All time-varying independent variable values were updated across the temporal span of each subject’s longitudinal records. We primarily employed factors other than combat duty and military occupation for inclusion in regression modeling to address possible confounding of their relationships with TCa. We created a dichotomous variable reflecting the presence of combat duty at each subject-specific observation over time. After such experiences, the variable retained its positive value for the duration of each affected subject’s subsequent records to account for the combat exposure history.

We identified military occupations based on the Army’s alphanumeric code system for job categories (U.S. Army, n.d.-b) that were present in administrative data. Occupation choices available at enlistment with the Army can be broadly divided into two groups. One group may be broadly termed the “combat arms,” comprising roles in which there is a focus on potentially employing lethal force.

Combat arms occupations include the infantry, a prevalent Army occupation that is considered the “backbone” of the force (Figgs, 2022). These soldiers physically carry most of their personal equipment and bear the primary responsibility for direct engagement with hostile forces. Infantry soldiers frequently maneuver on foot without the protection of armored vehicles or tanks, often relying on personally carried equipment to protect them against environmental and other threats. Other combat arms professions include “heavy” forces that operate armored vehicles such as tanks, artillery teams that support or operate large-caliber cannons, and Special Forces soldiers with unique skills and duties.

While all Army soldiers are trained in basic combat skills, another broad group of soldiers fulfill primarily supporting roles such as administrative, medical, and logistical jobs (U.S. Army, n.d.-b). Soldiers in supporting occupations together represent the largest total subgroup in the Army (Berry, 2021). Based on these ideas, we organized the study population in terms of occupations by using a categorical variable with three groups: infantry, other combat arms, and all military occupations other than combat arms.

Another possible TCa predictor of central interest was military service time, which we obtained from the official personnel records. We organized this factor in terms of months using continuous and four quantile-based categories. To address possible confounding, we organized further variables for statistical control. Because TCa incidence is highest among White men (Giona, 2022), and Army combat arms occupations may see higher proportions of White members than do other occupations, particularly in leadership roles (Lopez, 2016), the independent variables included a dichotomous term for White race versus all others. We arranged age in years as a continuous variable and in terms of four quantile-based groups. We created a variable accounting for socioeconomic status in terms of whether the subject was in the highest (i.e., officer) pay grades versus lower (enlisted) grades (U.S. Defense Finance and Accounting Service, 2023).

### Statistical Analysis

To present unadjusted descriptive data on possible associations between TCa and selected predictors, we tabulated independent variable statuses at the last person-specific observation for TCa cases and noncases. The resulting distributions were compared for statistically significant differences using chi-square tests of categorical variables. We additionally calculated two-sided *t*-tests comparing mean values of continuous variables for the cases and noncases. We computed TCa incidence rates for the total population and in terms of occupation- and military service time–based categories. We organized a figure graphically demonstrating TCa incidence rates for each subgroup.

To provide adjusted TCa risk estimates that accounted for possible confounding, we then computed a multivariable Weibull survival regression model against the dichotomous incident TCa outcome. All possible combinations of all of the selected independent variables were initially included in the model and the model development process included exploration of interaction terms as potential evidence of effect modification. Independent variables were eligible for the final model if (a) their presence resulted in at least a 10% change in at least one category of the variables of main interest (combat duty and military occupation) or (b) at least one category of the variable in question demonstrated statistical significance at the 95% confidence level. Fundamentally, we also required the combat duty and military occupation variables to meet the statistical significance criterion to be retained in the final model.

We applied a goodness-of-fit measure throughout the model iterations by confirming residuals that most closely approximated a standard exponential distribution in the case of the best survival model (Cox & Snell, 1968). When at least one variable selection criterion above was met, the measure further assisted in determining approaches such as whether continuous or categorical forms of applicable variables resulted in the best fit. Altogether, the factors included in the final model reported herein were those that survived the model selection process.

The regression model yielded adjusted hazard ratios or aHRs. The aHRs reflected the risk of a first-time TCa diagnosis associated with each independent variable as of each time point among the subjects who were as-yet undiagnosed by that time. The aHRs were interpretable as independent effect estimates for each variable when holding the other independent variables constant and comparing each status to a dedicated reference group.

The 95% confidence level was the threshold for statistical significance in all results. The analyses were conducted using Stata version 18.0 statistical software of StataCorp, College Station, Texas.

## Results

The study population consisted of 210,483 males who were followed for a total of 378,770.92 person-years. Individual mean follow-up time was 21.59 months (*SD*: 13.78 months; median: 21; range: 1–48). We observed 44 incident TCa diagnoses equating to a period prevalence of 0.02%. The overall incidence rate was 11.61 cases per 100,000 men per year. This figure was similar to incidence rates of 3.52 to 14.13 TCa cases per 100,000 person-years seen across similar age ranges in U.S. data (Centers for Disease Control and Prevention [CDC], 2024).

Table 1 presents the unadjusted comparisons of statuses at subjects’ final observations for the independent variables retained in the final regression model with organization by TCa case statuses. A history of combat deployment did not demonstrate a statistically significant unadjusted association with TCa. Infantry soldiers demonstrated the highest percentage of TCa cases by occupational subgroup (17 of 44 cases; 38.64%). The chi-square test for unadjusted TCa risk versus occupational category escaped statistical significance at the 95% confidence level by a small margin (*p* = .062).

**Table 1.** Subject Traits and their Distributions^a^ (*N* = 210,483) at the Last Person-Specific Observation for Those With and Without Incident Testicular Cancer (TCa) Diagnoses, With Statistical Tests for Distribution and Mean Differences.

Factor	Diagnosed with TCa?		Test result^b^
	No 210,439 (99.98)	Yes 44 (0.02)	
Deployed to combat			
No: 164,735 (78.27)	164,697 (78.26)	38 (86.36)	1.70 [0.193]
Yes: 45,748 (21.73)	45,742 (21.74)	6 (13.64)	
Occupation			
Infantry: 52,157 (24.78)	52,140 (24.78)	17 (38.64)	5.55 [0.062]
Other combat arms: 48,801(23.19)	48,790 (23.18)	11 (25.00)	
Other occupations: 109,525 (52.04)	109,509 (52.04)	16 (36.36)	
Military service time, months			
≤5: 40,686 (19.33)	40,677 (19.33)	9 (20.45)	18.48 [<0.001]
6–13: 33,381 (15.86)	33,365 (15.85)	16 (36.36)	
14–22: 44,778 (21.27)	44,767 (21.27)	11 (25.00)	
>22: 91,638 (43.54)	91,630 (43.54)	8 (18.18)	
Mean service time [standard deviation]	20.60 [13.78]	13.55 [10.16]	<0.001
Age, years			
17–19: 23,195 (11.43)	23,281 (11.06)	3 (6.82)	2.65 [0.449]
20–22: 130,772 (64.41)	57,119 (27.14)	9 (20.45)	
23–24: 49,050 (24.16)	55,031 (26.15)	12 (27.27)	
≥25:	75,008 (35.64)	20 (45.45)	
Mean age [standard deviation]	23.14 [3.66]	23.45 [3.02]	0.569
White race			
No: 54,555 (25.92)	54,553 (25.92)	2 (4.55)	10.47 [0.001]
Yes: 155,928 (74.08)	155,826 (74.08)	42 (95.45)	

a Format for counts and percentages: *n* (%). Cell counts and percentages in the top cell row sum horizontally for the total population. All other count and percentage data sum vertically. ^b^ Formats for distribution test results: Chi-square test results for differences in categorical variable distributions: chi-square statistic [*p* value].

Two-sided *t*-test of age mean differences: *p* value.

The highest overall TCa proportion by military service time was seen in the 6- to 13-month range. The mean service time at the final observation was lower for TCa cases, an expected finding given that cases were distributed across various time points, whereas most of the population without TCa was observed for the entirety of the 48-month data span. We saw no statistically significant differences in age versus TCa diagnosis status, whether organized categorically or if comparing means. By race, the vast majority of cases (*n* = 44; 95.45%; *p* < .001) occurred among White subjects.

**Figure 1.**
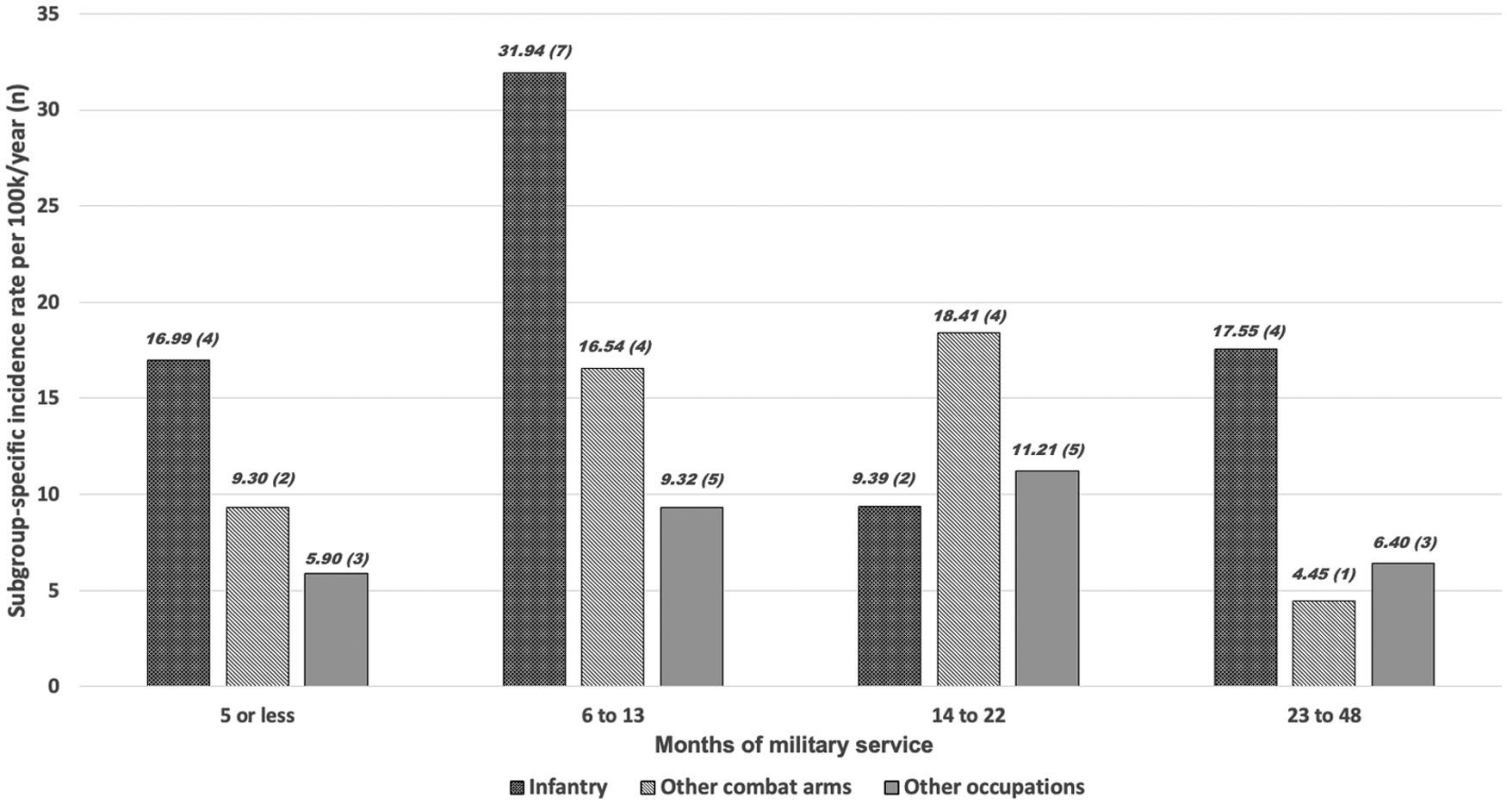
Testicular Cancer Rates Organized in Terms of Military Service Time-Based Groups and Military Occupations. Format: Case Rate (Case Count) Per Group.

When occupation and service time were organized together (Figure 1), TCa incidence rates for men in the infantry occupation demonstrated a notable peak in the range of 6 to 13 service months at 31.94 cases per 100,000 person-years. Across all occupations, more than one third (16 of 44; 36.36%) of the TCa cases occurred in this service time range. Infantry soldiers experienced the highest incidence rate in three of four service time categories. The sole exception was in the 14- to 22-month range, during which soldiers in other combat arms occupations saw the highest TCa rate at 18.41 cases. Most cases among infantry soldiers (11 of 17, 64.71%) occurred in the first 13 months of service.

Table 2 presents the results of the regression model providing control for the selected set of independent variables. The variables for combat deployment experience and military pay grades did not reach statistical significance at the 95% confidence level, did not demonstrate evidence of substantial confounding of other variables, and their inclusion did not result in increased goodness of model fit. Accordingly, these factors were not included in the final model.

**Table 2.** Results of a Multivariable Survival Regression Model for Incident Testicular Cancer Diagnoses in a Population of New U.S. Army Soldiers (*N* = 210,483).

Factor	Adjusted hazard ratio (aHR)	95% confidence interval for aHR	*p* value
Occupation (referent: other occupations)			
Infantry	2.02	1.01–4.02	.045
Other combat arms	1.47	0.68–3.18	.330
Military service time, months (referent: ≤ 5)			
6–13	7.23	2.75–19.00	<.001
14–22	4.40	1.51–12.79	.006
>22	2.09	0.64–6.81	.223
Age, years (referent: 17–19)			
20–22	1.46	0.38–5.60	.579
23–24	2.95	0.79–10.92	.106
≥ 25:	4.02	1.13–14.36	.032
White race (referent: no)			
Yes	6.82	1.64–28.39	.008

Subjects in the infantry occupation experienced an adjusted TCa risk that was 2.02 times as high as those in the reference group of all non-combat-arms occupations (95% confidence interval [CI]: 1.01–4.02; *p* = .045). Subjects with other combat arms professions did not demonstrate a statistically significant difference in adjusted TCa risk versus the referent. Service time of 6 to 13 years compared with 5 or fewer months demonstrated the highest overall aHR in the model (aHR: 7.23, *p* < .001). Age 25 or older conferred the highest TCa hazard by age (aHR: 4.02, *p* = .032) when compared with subjects 17 to 19 years old. The effect estimate for White race was nearly as high as that seen for service time of 6 to 13 months (aHR: 6.82, *p* = .008).

## Discussion

We did not observe increases in TCa risk associated with combat deployment in this population of new U.S. Army recruits. It is worth noting that combat service in this new-recruit study population necessarily occurred after completion of basic training and assignment to deployable units. The available follow-up time from observed combat service to the end of the data period may have limited the opportunities for post-deployment case detection. Altogether, the analysis does not rule out later, combat-related effects if follow-up were to continue.

The “healthy warrior” effect recognized in other contexts in military populations (Haley, 1998) also merits some thought when pondering the absence of a combat association. This concept suggests that the military’s rigorous standards and recurrent screenings reduce the chance of an individual with health problems deploying to combat. The phenomenon could artificially downregulate relationships between combat duty and later health outcomes.

Our careful use of time-series data in which predictors could not follow outcomes should have reduced the risk of this possibility. Toxic combat-related exposures including chemicals, metals, radiation, and “burn pits” are well-known, thus residual concerns about downstream TCa risk effects of combat duty beyond our observation duration remain. These issues have been deemed sufficiently common and concerning that the U.S. Congress has established dedicated prevention, research, and intervention programs to address them (DoD, 2024). More research using substantially longer follow-up than we could assess would be needed to rule out more remote associations with such exposures.

Men in the infantry profession saw a twofold adjusted risk increase compared with those in supporting professions. Notably, the variation was apparent within the maximum of 48 months of follow-up that were available in these data. An important takeaway from these findings is that they do not prove causation between any particular exposure and TCa. It is possible that individuals who select the infantry occupation also tend to possess unknown traits that are independently associated with TCa and for which we did not provide statistical control, resulting in unrealized confounding. A focused study is therefore required to ascertain whether the increased TCa hazard for infantry soldiers reveals a true causal relationship.

Such research may be challenging. Even when specific carcinogens appear well known, accurately establishing latency from initial exposure to tumor development can be difficult in cancer studies (Sloan et al., 2015). As discussed previously, there is relatively wide, exposure-dependent latency variation among known latency periods for solid tumors (Berrington de Gonzalez et al., 2012; Howard, 2015).

While latency in TCa could therefore be lengthy in at least some cases, there remains the potential for rapid cancer development in tissues with high cell turnover rates (Tomasetti & Vogelstein, 2015), which describes the testes. About 40% of non-seminomatous germ cell tumors of the testes are aggressive and fast-growing (Johns Hopkins Medicine, n.d.). Altogether, it appears questionable whether progression from novel exposures to diagnosable TCa could occur during the time intervals we observed, but it cannot be ruled out using our data. Because of the potential for our findings to reveal a causal relationship, the importance of continued research to identify or rule out occupational TCa predictors appears evident.

Our findings suggest some initial targets for research seeking to identify specific carcinogens associated with military professions. Infantry soldiers frequently train with firearms. Consequently, they could see disproportionally high exposure to heavy metals and chemical fumes that are known to be produced by small-arms gunfire compared with other soldiers. These compounds readily permeate lung tissues and could be systemically absorbed (Mariussen et al., 2021).

A range of environmental exposures could merit study due to the frequent ambulatory activity seen in this population. Such activity could generally increase risks of exposure to airborne toxins when compared with soldiers who mainly operate in vehicles or tanks with air filtration systems. Infantry forces may also need to physically ford bodies of water during dismounted operations. This activity could result in immersive exposures to carcinogenic chemicals from sources including industry and agriculture (Gidley, 2022).

When holding occupation, age, and race constant, military service in the range of 6 to 13 months was associated with the highest adjusted TCa diagnosis risk. The adjusted hazard dwindled with time thereafter (Table 2). One possible explanation for this prevalent diagnosis timing is that Army soldiers are required to undergo annual health screenings (U.S. Military Health System, 2023). The first such screening at around 1 year of service would provide the first opportunity to detect serious health conditions.

Altogether, the data support the potential for a screening effect in which accumulated cases in the population are highly likely to be detected at the first screening and cases are progressively exhausted thereafter. The TCa hazard increases seen with increasing age within the relatively limited age range of this population and with White race are consistent with expectations from the prior literature. We found no evidence of short-term TCa risk associated with military pay grade.

In terms of strengths, the study leveraged standardized data on a large population in which a reasonably substantial number of TCa cases were present. The analysis was generally valuable for demonstrating the importance of conducting multivariable analyses to rule out confounding effects. Occupation bordered on but did not reach statistical significance in the unadjusted analyses. The regression model was required to reveal the statistically significant, independent effect of occupation after applying control for the other independent variables. This phenomenon underlines the dangers associated with basing surveillance and associated policies on unadjusted analyses of raw statistics.

The study was potentially limited by the use of diagnosis code data that can be subject to imprecision. The seriousness of TCa diagnoses could confer a lower risk of such problems than might be seen with other outcomes. Otherwise, the analysis was limited by the time available for follow-up. External generalizability may be greatest for other U.S. military branches, the military members of other nations, or other uniformed groups with similar exposures.

While the aforementioned military service entry requirements were reassuring with respect to detecting incident TCa, we were unable to specifically examine the medical or other histories of the subjects preceding their military service. Unavailable exposure data included the geographic regions in which the subjects were recruited and/or resided in their childhood and teenage years. Prior research found that regional differences in Europe did not predict TCa risk (Sloan et al., 2015), reducing some concerns about this limitation. Regional TCa risk variation in the United States nonetheless deserves increased attention in future research.

In conclusion, the study suggests that combat duty was not associated with TCa risk elevation, but risk was higher for new infantry soldiers than in supporting occupations. Recognizing the uncertainty regarding the causal pathway for the findings by occupation, the information could be useful for recruits in their career planning and as part of the military’s preventive efforts. We call on governments to correct the inadequate TCa funding when compared with other cancer types. The research community should investigate TCa rates and predictors in military populations to identify actionable preventive and management opportunities, particularly if access to much longer time spans in retrospective data becomes possible.
